# A long noncoding RNA positively regulates CD56 in human natural killer cells

**DOI:** 10.18632/oncotarget.12466

**Published:** 2016-10-04

**Authors:** Ruya Zhang, Fang Ni, Binqing Fu, Yang Wu, Rui Sun, Zhigang Tian, Haiming Wei

**Affiliations:** ^1^ Institute of Immunology and the CAS Key Laboratory of Innate Immunity and Chronic Disease, School of Life Science and Medical Center, University of Science and Technology of China, Hefei National Laboratory for Physical Sciences at Microscale, University of Science and Technology of China, Hefei, China; ^2^ Department of Pathophysiology, Anhui Medical University, Hefei, Anhui, China

**Keywords:** long noncoding RNAs, natural killer cells, CD56, primary lymphocytes, lnc-CD56, Immunology and Microbiology Section, Immune response, Immunity

## Abstract

Natural killer (NK) cells are innate immune lymphocytes that play critical roles in host defense against viral infection and surveillance against malignant transformation. Long noncoding RNAs (lncRNAs) are important immune system regulators. Here, we analyzed human primary lymphocyte lncRNA expression profiles to identify NK-lncRNA signatures. We detected numerous novel NK-specific lncRNAs with potential roles in regulating human NK cell differentiation and function. Expression of lnc-CD56, an NK-specific lncRNA, was positively correlated with that of CD56, a classical human NK cell surface marker. We showed that lnc-CD56 may function as a positive regulator of CD56 in primary human NK cells and differentiated NK cells from human CD34^+^ hematopoietic progenitor cells. Our data provide an annotated human NK cell lncRNA expression catalog and demonstrate a key role for lncRNAs in NK cell biology.

## INTRODUCTION

NK cells are an important component of the innate immune system, providing early host defense against viruses and invading pathogens, and contributing to the early detection and destruction of transformed cells [1-5]. In humans, NK cells are classified as being positive for CD56 and negative for CD3, without rearranged T-cell receptors [6]. NK cells development and function is likely dependent on a synchronized set of transcriptional and posttranscriptional events [7-9]. In addition to the well-established role of transcription factors as instructive signals within the NK cell molecular program, noncoding RNAs (ncRNAs) are emerging as new NK cell regulators. Among the ncRNAs classes that play roles in immune cell biology, microRNAs (miRNAs) are currently the best characterized. miRNAs directly impact NK cell development, cytokine production and cytotoxicity [10]. We previously reported that miR-483-3p plays a critical role in the cytotoxic activity of human NK cells [11], and identified miR-362-5p as an essential regulator of NK cell global function *via* miRNA array analysis [12]. NK cell development and global functions have been thoroughly investigated through comprehensive gene and miRNA expression analyses [12-14], and several transcription factors and miRNA families of short ncRNAs have been identified [7, 10]. However, whether or not long noncoding RNAs (lncRNAs) play a role in NK cell biology is largely undetermined.

Long noncoding RNAs (lncRNAs) are RNA transcripts longer than 200 nucleotides that do not encode proteins [15, 16]. lncRNA expression is tissue-specific, and expression changes in a tissue have been associated with various human illnesses, including cancer, inflammation and neurological diseases [17-19]. lncRNAs reportedly play crucial roles in the immune system [20-22]. For example, lncRNA expression is correlated with differentiation and activation of immune cells, including T cells, B cells, macrophages and DCs cells [23-29]. However, few recent functional lncRNAs have been described in NK cells, and little is currently known about lncRNAs that affect expression of human NK cell-regulating genes.

Recent studies have also begun to define lncRNAs expressed by human T and B lymphocytes at varying development and differentiation stages [30, 31]. Such genome-wide analyses aim to identify functional, lineage-specific lncRNAs, and highlight the relevance of lncRNAs in regulating immune responses. Here, we broadly analyzed lncRNA expression in three different highly purified human NK cell populations and identified NK cell-specific lncRNA signatures. Specifically, we focused on lncRNAs upregulated in human NK cells as compared to T cells, and identified a novel lncRNA, lnc-CD56, that positively regulates CD56 on human NK cells. Collectively, our analyses identified novel NK-specific lncRNAs and showed that lnc-CD56 regulates CD56 expression in human NK cells. Our present study constitutes the first comprehensive inventory of human NK cell lncRNAs and demonstrates that lncRNAs can be critical to NK cell-specific phenotypes and functions.

## RESULTS

### Identification of lncRNA signatures in human primary NK cells and T cells

**Figure 1:**
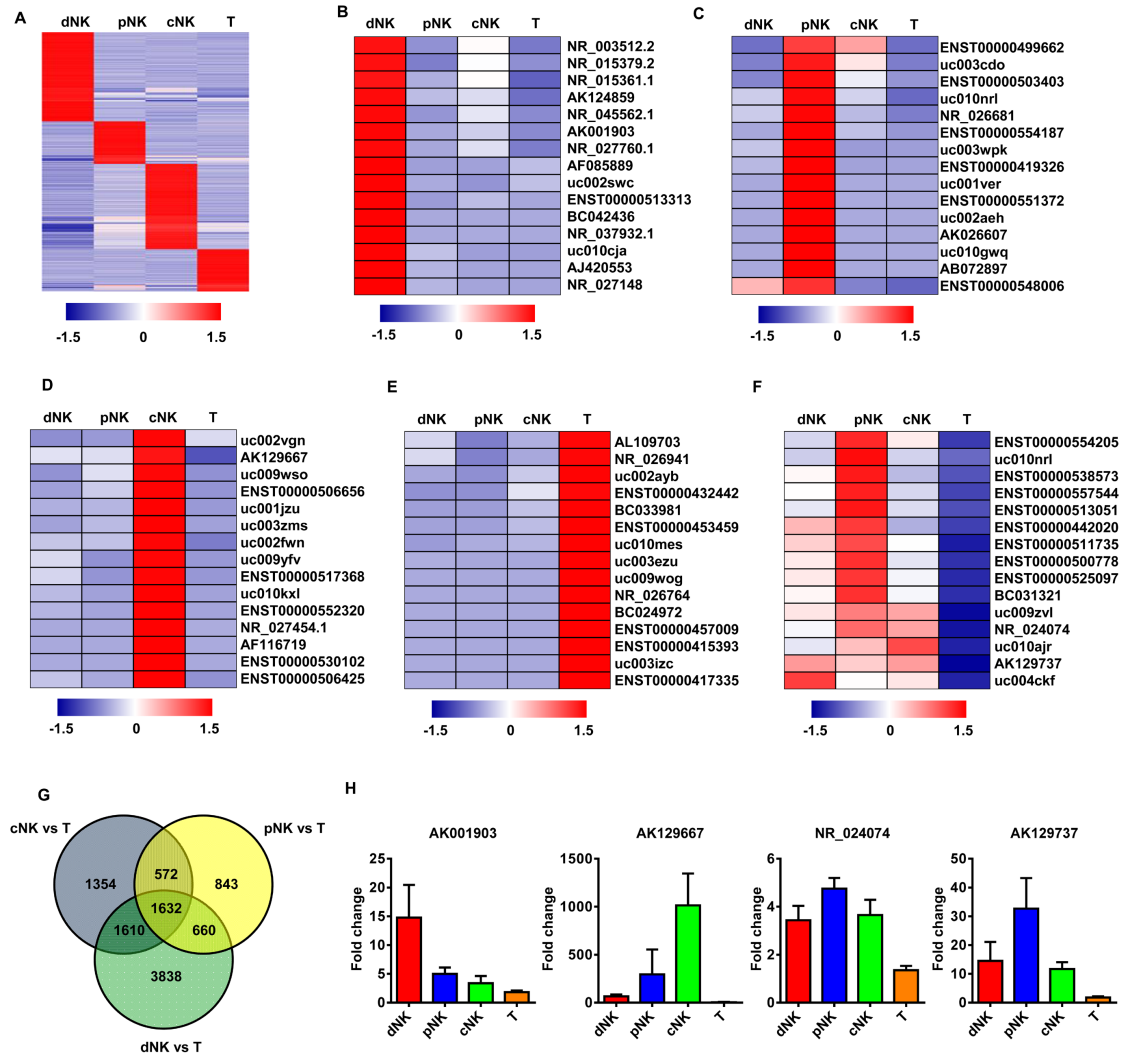
lncRNAs expression profiling in primary human lymphocyte subsets Heat map of normalized expression values of signature lncRNAs in human dNK, cNK, pNK, and T cells selected on the basis of fold change ( > 2 with respect to all other subsets) **A.** Heat maps of the top 15 signature lncRNAs upregulated in dNK **B.**, cNK **C.**, pNK **D.** and T cells **E.** derived from the microarray data. Rows: an individual lncRNA; columns: an individual cell subset. Blue and red pseudocolors indicate transcript levels below and above the mean, respectively. The top 15 upregulated, NK-specific lncRNAs with > 2-fold change in the given comparisons (dNK *vs*. T; cNK *vs*. T; and pNK *vs*. T) **F.** Venn diagram showing the number of all differentially expressed lncRNAs across the following comparisons: dNK *vs*. T, cNK *vs*. T, pNK *vs*. T **G.** The number of differentially expressed lncRNAs from each comparison is indicated. Validation of NK signature lncRNA expression by qRT-PCR with purified human dNK, cNK, pNK and T cells from healthy donors (average of three independent experiments) **H.**

We used previously developed methods [11, 12] to purify human NK cells from periph­eral blood (pNK), cord blood (cNK), uterine deciduas (dNK) and T cells from periph­eral blood (used as controls). We then examined the lncRNA expression profiles in these purified lymphocyte subsets through transcriptome microarray analysis. For this analysis, authoritative data sources containing more than 38,942 lncRNAs were utilized. Expression profiles of 7,382 lncRNAs indicated that each lymphocyte population was characterized by a distinct lncRNA signature (Figure 1A).

As lncRNAs are generally more cell-specific than protein-coding genes [17, 18], we focused on the subset of lncRNAs exhibiting NK cell-specific expression. We searched for human NK cell-specific lncRNAs in the entire data set, and identified the top 15 differentially expressed lncRNAs (ranked by expression fold change between NK and T cells) that had greater than two-fold expression differences in a given lymphocyte subset compared to all other subsets (Figure 1B-1E). We also selected differentially expressed lncRNAs that had more than a two-fold expression difference in a particular comparison (dNK *vs*. T; cNK *vs*. T; or pNK *vs*. T). These stringent criteria were met by 1632 lncRNAs that were specifically up- or downregulated in NK cells as compared to T cells (Figure 1F-1G). To verify the microarray data, four of these lncRNAs were selected for real-time PCR validation. qRT-PCR confirmed the high expression of these lncRNAs in various human NK cell populations (Figure 1H).

### Identification of lncRNA signatures in human NK populations

**Figure 2:**
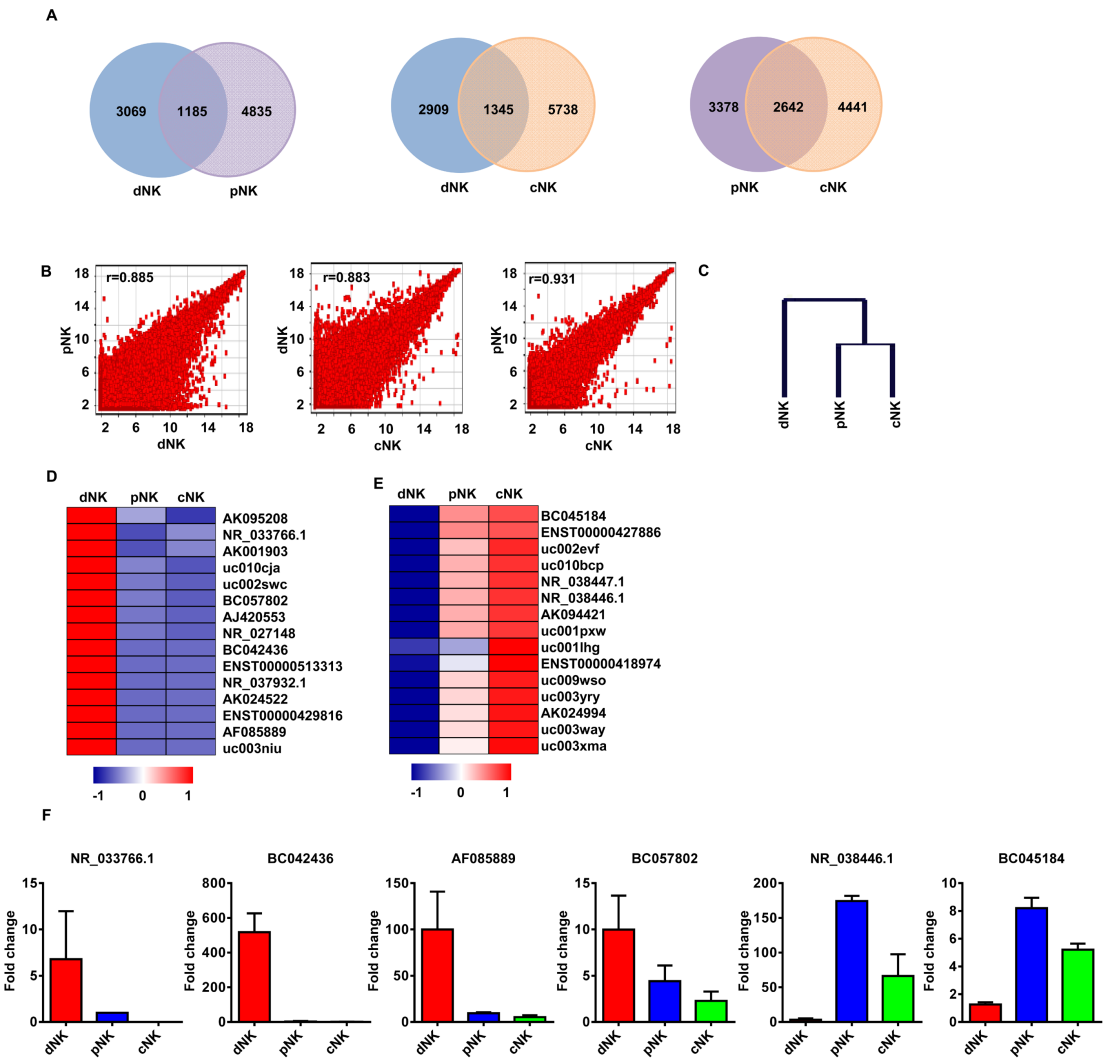
LncRNA signatures in various human NK populations Shared lncRNAs in NK populations: dNK-pNK, dNK-cNK, cNK-pNK **A.** Log base 2_intensity plots of lncRNA levels for dNK, cNK, and pNK cells **B.** Axis labels are log 2 scaled. Pearson Correlation r values were used to establish the linear fit of the data. Hierarchical tree of dNK, cNK, and pNK cells by clustering analysis with lncRNA microarray data **C.** The top 15 lncRNAs up- and downregulated in dNK cells when compared to cNK and pNK cells based on hierarchical clustering are shown in **D.** and **E.**, respectively. Quantitative RT-PCR analysis of randomly selected lncRNAs from **D.** and **E.** in human dNK, cNK and pNK cells **F.** Data represent three independent experiments.

We first employed a Venn diagram to illustrate the lncRNAs shared between human primary NK cell populations from different cell compartments (e.g., dNK-pNK, dNK-cNK, and cNK-pNK). Although most of the lncRNAs were co-expressed in different human NK cell populations, pNK and cNK cells shared more lncRNAs (2642) than dNK and pNK (1185), or dNK and cNK cells (1345) (Figure 2A). We also performed a Pearson correlation scatter plot analysis among dNK, cNK and pNK cells. Two comparisons (dNK-pNK and dNK-cNK) produced r < 0.9, indicating that the dNK cell lncRNA profile was distinct from that of pNK and cNK populations (Figure 2B). However, the results showed high similarities between cNK and pNK populations (r = 0.931, Pearson's correlation). These relationships between distinct NK cell populations were further confirmed through unsupervised hierarchical clustering analysis of lncRNA profiles (Figure 2C).

To further differentiate dNK cell lncRNA profiles from those of the other NK cell populations, we identified those lncRNAs from the above analyses that were preferentially up- or downregulated in dNK cells by hierarchical clustering analysis (Figure 2D-2E and S2). We selected the top 15 lncRNAs up- or downregulated in dNK as compared to cNK and pNK cells (Figure 2E-2F). To validate the microarray data, we selected four lncRNAs (NR_033766.1, BC042436, AF085889 and BC057802) upregulated in dNK cells and two (NR_038446.1 and BC045184) downregulated for qRT-PCR confirmation. Similar variations in the selected lncRNAs were found between microarray and qRT-PCR analysis data (Figure 2F). Taken together, these data demonstrate that lncRNAs provide specific signatures for various human NK populations, which may contribute to distinct phenotypes and functions for NK cells from different cell compartments.

### Identification of potentially functional lncRNAs in human NK cells

**Figure 3:**
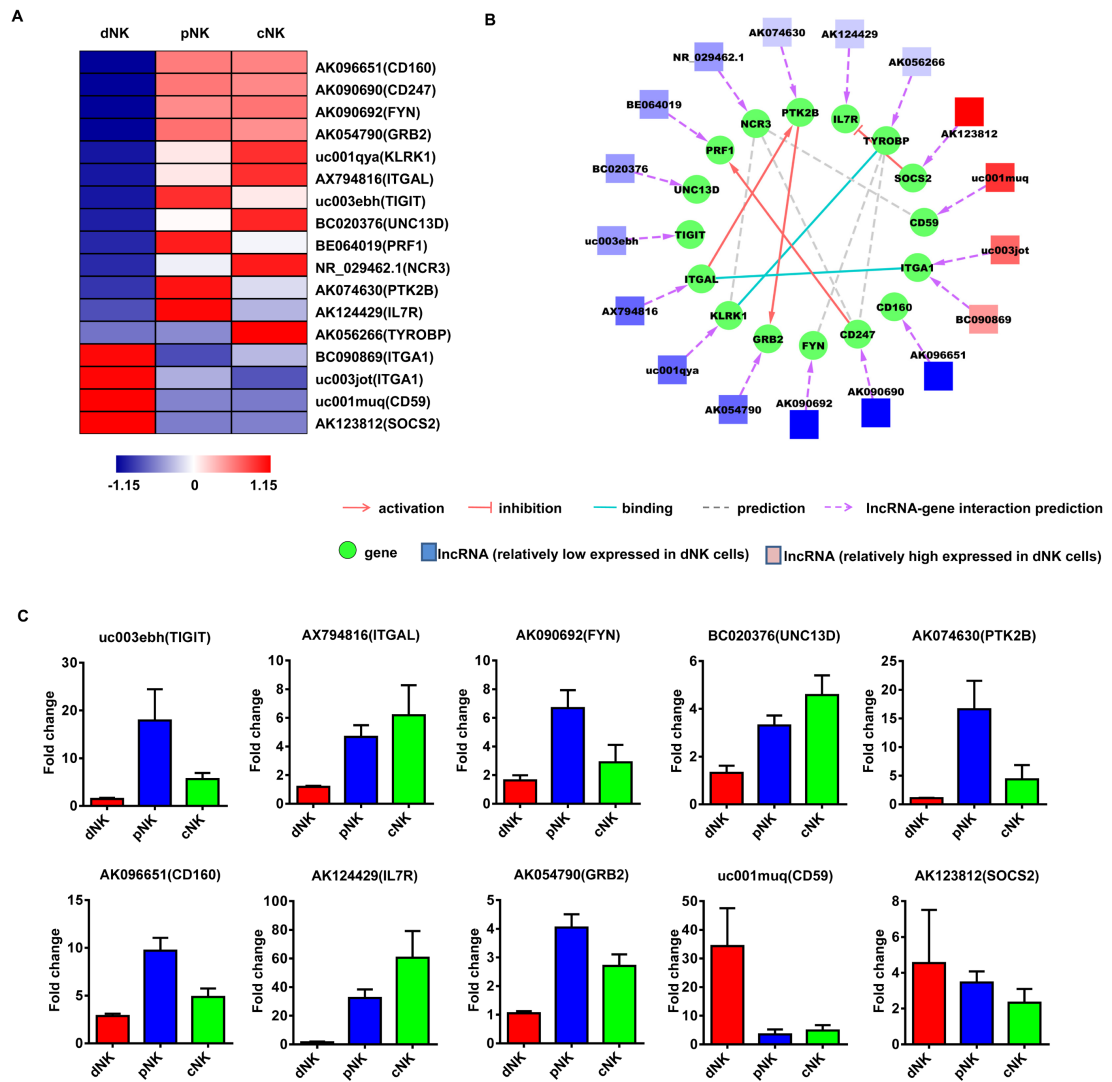
Identification of potentially functional lncRNAs involved in NK cell cytotoxic activity Heat map of lncRNAs with predicted target genes involved in NK cell cytotoxic activity **A.** Displayed lncRNAs were up- or downregulated at least 2-fold in pNK and cNK cells relative to dNK cells. Predicted interaction network among candidate cytotoxicity-related lncRNAs and protein-coding genes **B.** Square nodes: lncRNAs; round nodes: protein-coding genes. Verification of lncRNAs identified in **A.** by real-time PCR **C.** 18S rRNA was used as an internal control. Data represent three independent experiments.

**Figure 4:**
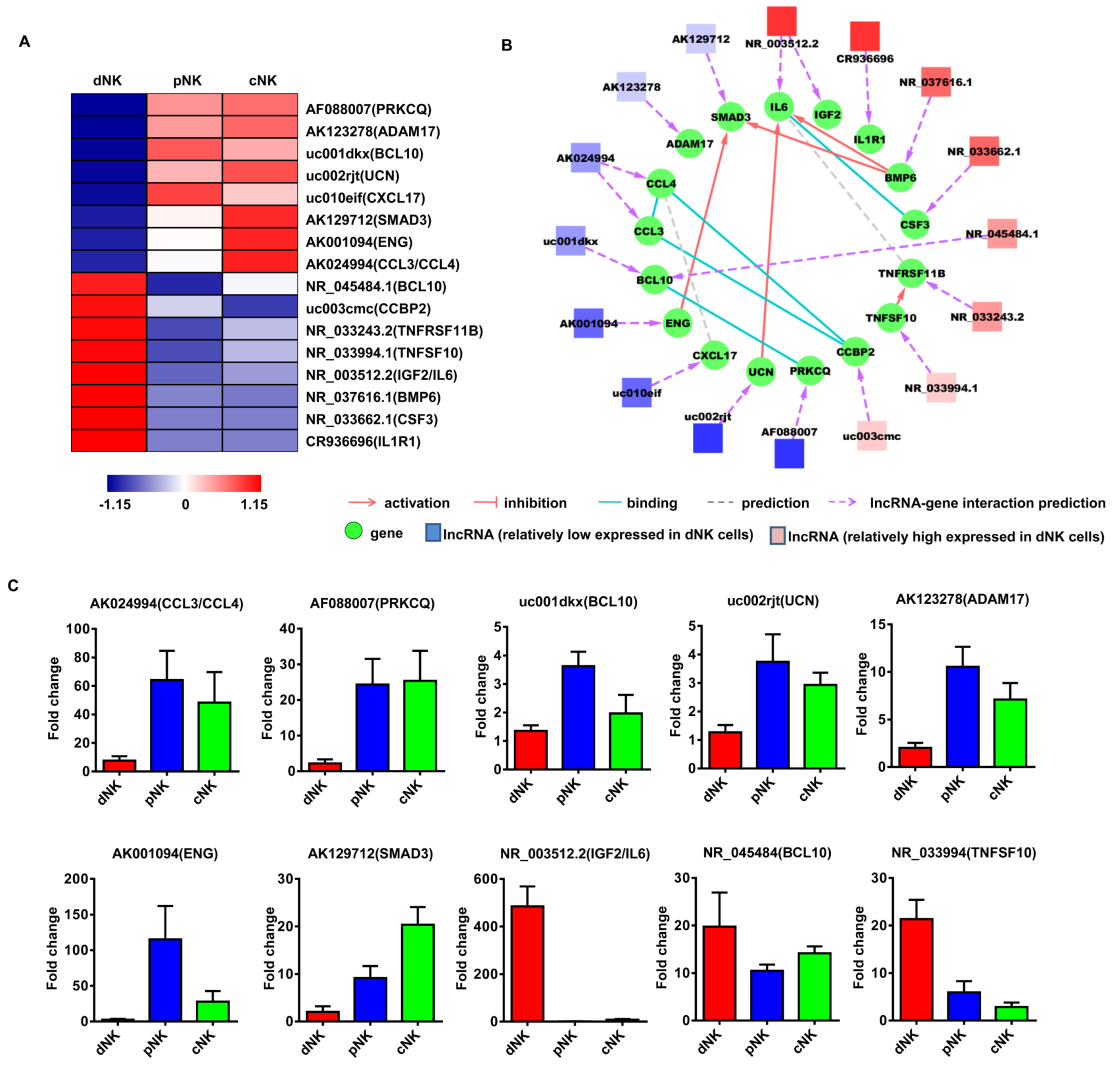
Identification of potentially functional lncRNAs involved in NK cell cytokine secretion Heat map of lncRNAs with predicted target genes involved in cytokine secretion **A.** Displayed lncRNAs were up- or downregulated at least 2-fold in pNK and cNK cells relative to dNK cells. Predicted interaction network among candidate cytokine secretion-related lncRNAs and protein-coding genes **B.** Square nodes: lncRNAs; round nodes: protein-coding genes. Verification of lncRNAs identified in **A.** by real-time PCR **C.** 18S rRNA was used as an internal control. Data represent three independent experiments.

**Figure 5:**
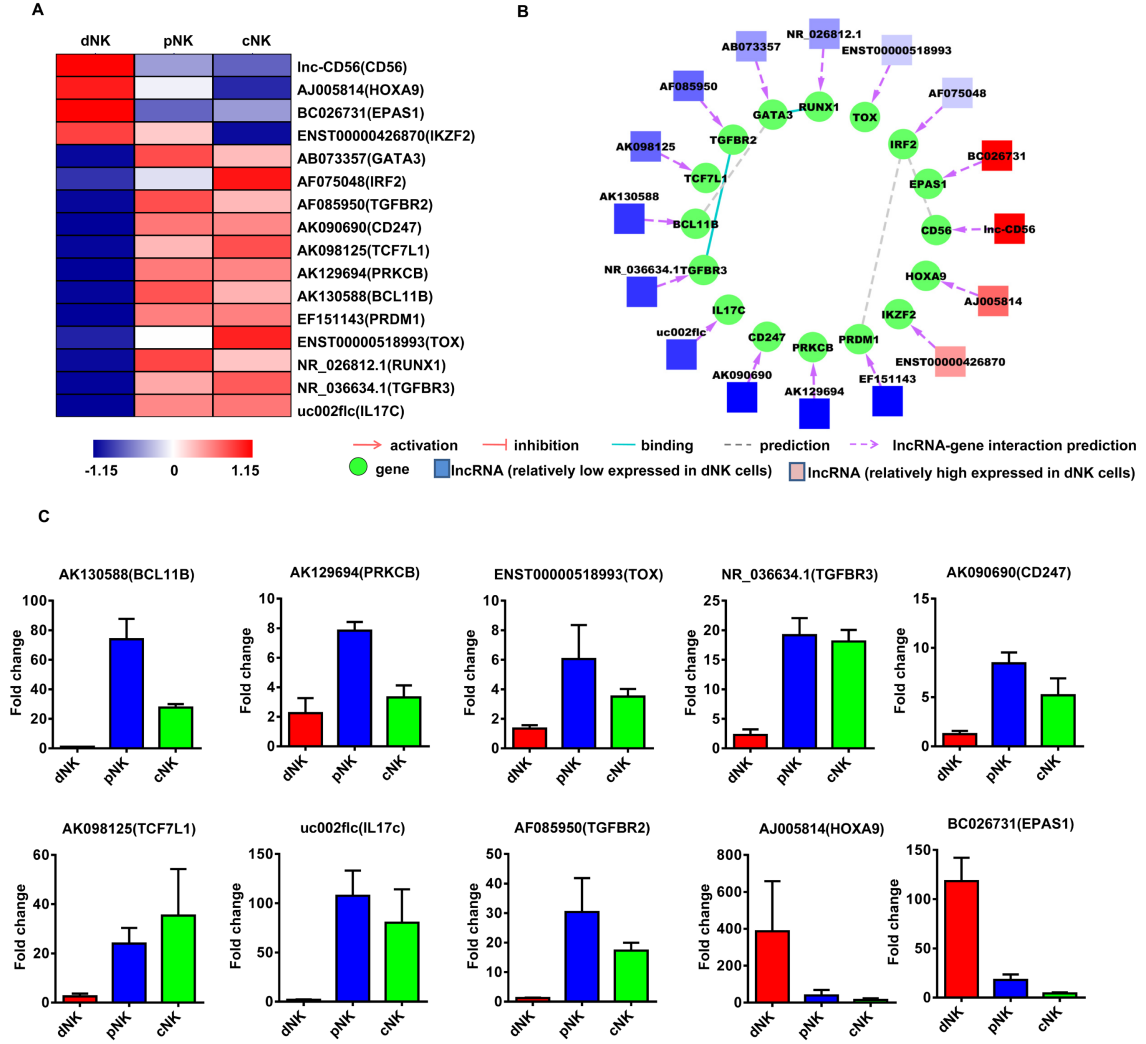
Identification of potentially functional lncRNAs involved in NK cell differentiation Heat map of lncRNAs with predicted target genes involved in NK cell differentiation **A.** Displayed lncRNAs were up- or downregulated at least 2-fold in pNK and cNK cells relative to dNK cells. Predicted interaction network among candidate cytokine secretion-related lncRNAs and protein-coding genes **B.** Square nodes: lncRNAs; round nodes: protein-coding genes. Verification of lncRNAs identified in **A.** by real-time PCR **C.** 18S rRNA was used as an internal control. Data represent three independent experiments.

To investigate the potential functions of these NK-associated lncRNAs, we input the predicted lncRNA target genes into the Database for Annotation, Visualization and Integrated Discovery [32, 33] (DAVID; http://david.abcc.ncifcrf.gov/), which utilized Gene Ontology (GO) to identify gene molecular function. Thus, we carried out lncRNA classification and subgroup analysis to associate these differentially expressed lncRNAs with their predicted target genes. After lncRNA classification into functional categories, such as cytotoxicity (Figure 3A), cytokine production (Figure 4A) and differentiation (Figure 5A), we superimposed lncRNA target predictions onto the lncRNA-mRNA correlation network based on genomic co-location and co-expression of lncRNAs and protein-coding genes (Figure 3B, 4B and 5B). These results further associated potentially functional lncRNAs with direct regulation of target mRNAs, suggesting that such lncRNAs are likely involved in human NK cell differentiation and/or function.

Finally, to confirm the lncRNA microarray results, we randomly selected several lncRNAs for qRT-PCR verification in a new set of primary human dNK, cNK and pNK cell samples (Figure 3C, 4C and 5C). qRT-PCR analyses confirmed lncRNA levels in human NK cells and were consistent with our lncRNA microarray data. The above results indicated that human NK cell subtypes exhibit unique lncRNA signatures.

One of the goals of this work was to identify novel lncRNAs that may contribute to the specific phenotypes and functions of human NK cells. From the above established lncRNA-mRNA interaction networks, we highlighted two novel lncRNAs —AK096651 and AB128931. AK096651 is predicted to target CD160, which is associated with the CD56^dim^CD16^+^ cytotoxic NK cell phenotype and is essential forNK-mediated IFN-γ production [34, 35]. This implies that the AK096651 might be involved in CD160 regulation, contributing to the human NK cell effector function. We also found that AB128931 expression was higher in human dNK cells compared with pNK and cNK cells, and was predicted to target CD56 (NCAM1). CD56 is a prototypical human NK cell marker and is involved in NK cell differentiation and development [36]. We hypothesized that AB128931 might contribute to a specific phenotype as well as the development of human NK cells.

Human NK cells are classified into CD56^bright^ and CD56^dim^ subsets based on CD56 and CD16 (FcγRIIIa) expression, and these distinct subsets differ in phenotype, function and tissue localization [37]. lncRNA expression profiles of freshly isolated CD56^bright^ and/or CD56^dim^ NK cells from decidua, cord blood and peripheral blood were compared using lncRNA microarrays (Figure S3A). We examined the lncRNA expression profiles of CD56^bright^ dNK, CD56^bright^ pNK and CD56^bright^ cNK cells. Hierarchical clustering indicated that the lncRNA expression profiles of CD56^bright^ dNK, CD56^bright^ pNK and CD56^bright^ cNK populations showed high similarities (Figure S3B). We also performed lncRNA classifications and subgroup analyses to associate differentially expressed lncRNAs with their predicted target genes, and to identify differences between CD56^bright^ cNK, CD56^bright^ pNK, CD56^dim^ cNK and CD56^dim^ pNK cells (Figure S3C). We found that lncRNA AB128931 was upregulated in CD56^bright^ as compared with CD56^dim^ subsets in both human decidua and peripheral blood (Figure S3D). These lncRNA profiles identified differences between dNK, cNK and pNK cells, and between the CD56^bright^ and CD56^dim^ subsets.

### Lnc-CD56 is highly expressed in human NK cells

**Figure 6:**
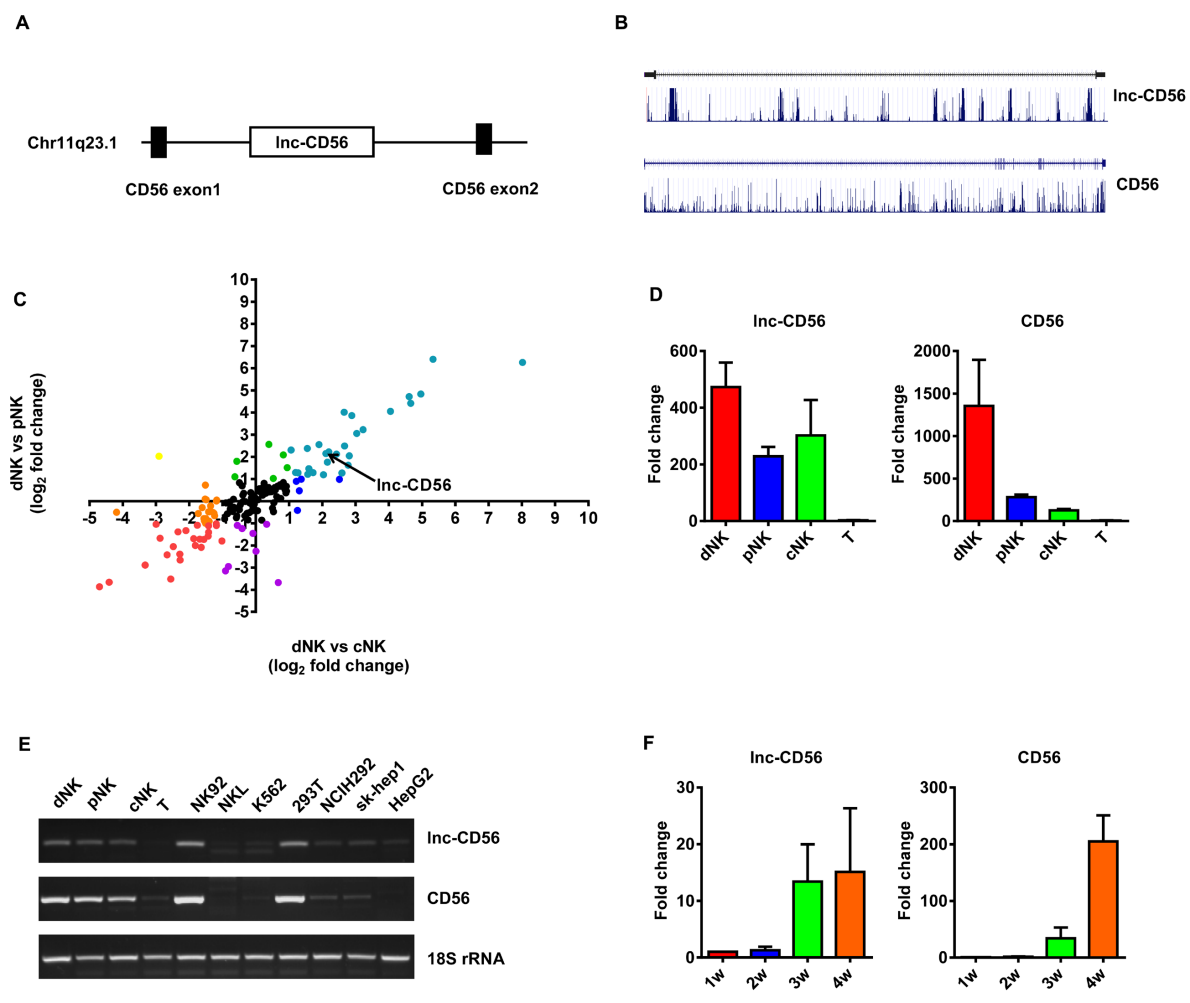
lnc-CD56 is highly expressed in human NK cells lnc-CD56 and CD56 location analysis **A.** lnc-CD56 is less conserved than *CD56* **B.** Scatter plots of log base 2 fold changes of lncRNAs targeting clusters of differentiation (CD) molecules in different cell types **C.** Each plot represents one lncRNA. Each color represents one expression type. Quantitative RT-PCR validation of lnc-CD56 expression in dNK, cNK, pNK and T cells **D.** 18S rRNA was used as an internal control. Semi-quantitative PCR analysis of lnc-CD56 expression in various human primary lymphocytes and cell lines **E.** Quantitative RT-PCR analysis of lnc-CD56 expression in cytokine-differentiated CD34^+^ cells at the indicated times **F.** Data represent three independent experiments.

As the above results suggested that lncRNAs may participate in the differentiation and effector function of human NK cells (Figure 3-5), to further identify a specific lncRNA with a critical role in human NK cells, we focused on the lncRNA AB128931, which is differentially expressed across various human NK populations (Figure 5A and S3D) and is predicted to target CD56, a prototypical NK cell marker. AB128931 is also called lnc-CD56, because this lncRNA is located within the first intron of *CD56* (Figure 6A). Bioinformatics analysis of lnc-CD56 revealed that it is not a protein-coding gene (Coding Potential Calculator; http://cpc.cbi.pku.edu.cn/programs/run_cpc.jsp) [38]. Gene conservation analysis indicated that lnc-CD56 is less conserved than CD56 (UCSC Genome Browser Comparative Genomics for conservation analysis; https://genome.ucsc.edu/) (Figure 6B). Microarray results also showed that lnc-CD56 was upregulated in dNK cells as compared to pNK and cNK cells (Figure 6C). We confirmed the high expression of lnc-CD56 in human dNK cells by qRT-PCR (Figure 6D). Higher lnc-CD56 expression correlated with larger amounts of *CD56* transcript in dNK cells; conversely, T cells exhibited very low lnc-CD56 expression and almost no *CD56* transcripts (Figure 6D-6E).

As we and others reported previously, NK cells can be differentiated from human umbilical cord blood (UCB)-derived CD34^+^ hematopoietic progenitor cells (HPC) when cultured *in vitro* with SCF/Flt3-L/IL-15-containing media for 28 d [11, 39]. Therefore, we assessed lnc-CD56 and CD56 expression in UCB/CD34^+^-derived NK cells cultured for 28 d. As expected, lnc-CD56 and CD56 expression positively correlated in human CD34^+^ HPC-derived NK cells *in vitro.* In human CD34^+^ HPCs differentiating towards NK cells, both lnc-CD56 and CD56 were poorly expressed for the first 14 d, but then increased continually up to 28 d (Figure 6F). These results indicated that lnc-CD56 was highly expressed in human primary NK cells and may constitute a specific NK cell marker.

### Lnc-CD56 is a positive regulator of CD56 in human NK cells

**Figure 7:**
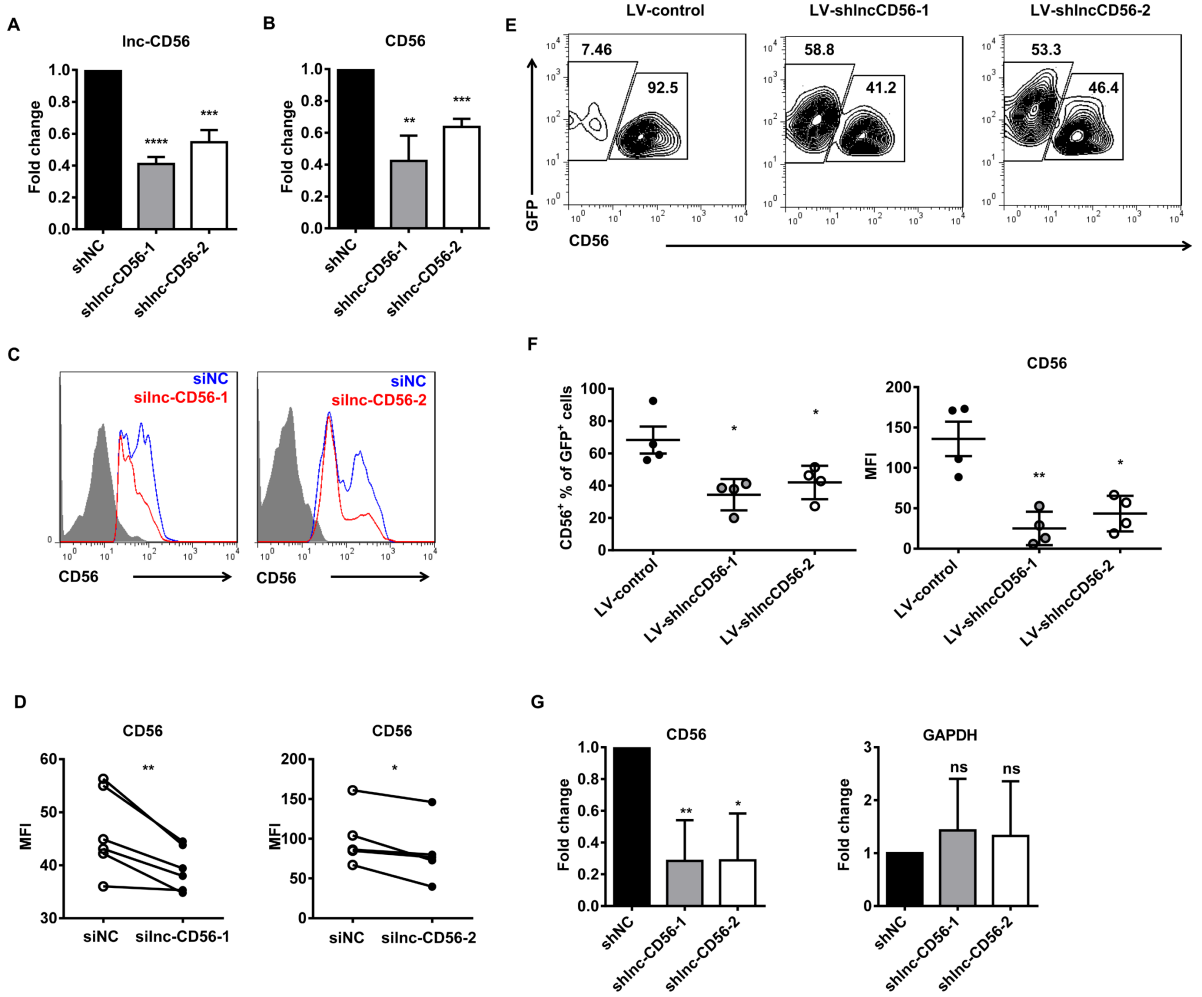
lnc-CD56 positively regulates CD56 in human NK cells qRT-PCR assay of lnc-CD56 and CD56 in HEK-293T cells transfected with lnc-CD56 shRNAs and control shRNA (shNC) shown in **A.** and **B.** respectively. Flow cytometry for CD56 expression in human dNK cells transfected with lnc-CD56 siRNAs (red line) or control siRNA (siNC, blue line) **C.** Mean fluorescence intensity (MFI) of CD56 expression from **C. D.** RT-PCR and flow cytometry were performed 24 h post-transfection. Purified CD34^+^ cells were transduced with lentiviral vectors overexpressing lnc-CD56 antisense shRNAs (LV-shlnc-CD56) or negative control shRNA (LV-control), and cultured for 4 weeks in NK cell differentiation conditions **E.** Representative flow-cytometry analysis of CD56 expression in the above cultured cells at the indicated times. Average CD56^+^ cell relative frequency and CD56 expression mean fluorescence intensity (MFI) in **E.** at 4 weeks **F.** Nuclear run-on experiments confirmed that lnc-CD56 knockdown reduced CD56 expression **G.** GAPDH was used as a negative control. Data represent of three independent experiments. *P < 0.05, **P < 0.01, ***P < 0.001, ****P < 0.0001 (Student's *t*-test).

Given that higher lnc-CD56 expression positively correlated with CD56 expression in dNK cells (Figure 6C-6D), we hypothesized that lnc-CD56 may function as a positive regulator of CD56 in human NK cells. We studied the role of lnc-CD56 in modulating CD56 expression using an shRNA-based loss-of-function approach. lnc-CD56 knockdown HEK-293T cells (transfected with lnc-CD56 pMSCV-shRNA) exhibited decreased CD56/NCAM1 mRNA levels (Figure 7A-7B). Similar results were observed in flow cytometry analysis of surface CD56 on dNK cells after transfection with lnc-CD56 siRNAs (Figure 7C-7D), confirming that lnc-CD56 regulates CD56 expression.

We next assessed the role of lnc-CD56 in the *in vitro* differentiated NK system. Validated scramble and lnc-CD56 knockdown lentiviral constructs were transduced separately into primary CD34^+^ HPCs, which were subsequently differentiated along the NK cell lineage for four weeks. Similar to human uterine dNK cells, lnc-CD56 knockdown decreased the percentage of mature CD56^+^ NK cells, as well as the mean fluorescence intensity (MFI) of CD56 expression in these CD34^+^-derived NK cells (Figure 7E-7F).

Considering that lnc-CD56 is located within the *CD56* intron (Figure 6A), it is possible that lnc-CD56-targeing shRNA directly bound to *CD56* pre-mRNA, leading to its degradation and reducing mRNA level. To exclude this possibility, we performed nuclear run-on experiments to further examine whether lnc-CD56 knockdown directly affected CD56 transcript level. We found that lnc-CD56 knockdown reduced CD56 transcription (Figure 7G). This observation indicated that reduced CD56 expression after lnc-CD56 knockdown was not due to the direct effect of shRNA on CD56 pre-mRNA, but the impaired stability of CD56 transcript. The above results show that lnc-CD56 functions as a positive regulator of CD56.

## DISCUSSION

Long noncoding RNAs are a newly-identified class of transcripts in the genome [40]. Despite the importance of lncRNAs in the immune system, it is not yet clear whether lncRNAs are involved in human NK cell regulation. The purposes of the present work were to: 1) employ genome-wide analyses to determine the scope of NK cell-specific lncRNA expression; and 2) identify novel functions of NK cell-specific lncRNAs.

Human NK cell populations from different tissues differ in phenotype and function [37]. However, the mechanisms behind such differences are not yet well understood. In addition, current knowledge of the molecular mechanisms that govern the onset and maintenance of specific human NK cell phenotypes is still incomplete. Our previous work indicated that pNK and dNK cells differ in their patterns of gene and microRNA expression [12, 13]. The present study extends this comparison to long noncoding RNAs among different human NK cell populations. A comprehensive lncRNAs expression landscape in three distinct human primary NK cell populations (pNK, cNK and dNK), as well as T cells from human blood, is presented. lncRNA profiling analysis identified numerous lncRNAs selectively expressed in different human primary NK populations. Multiple complex interaction networks were constructed based on genomic co-location and co-expression of lncRNAs and protein-coding genes. The majority of these co-expressed genes encoded proteins with NK-related immunologic functions, including cytotoxicity, cytokine secretion and differentiation. For this reason, it is likely that these lncRNAs regulate the transcription of neighboring or adjacent co-expressed protein-coding genes, playing a critical role in regulating the development and diverse functions of human NK cells. However, this must be further explored experimentally.

In the present lncRNA array-based study, we identified a novel lncRNA, lnc-CD56, that is more highly expressed in human decidual CD56^bright^NK cells than peripheral blood CD56^dim^NK cells. We therefore hypothesized that lnc-CD56 levels in human NK cells could be important for acquisition and maintenance of the CD56 surface marker occurs during NK cell development. We found that lnc-CD56 knockdown reduced CD56 expression in human decidual CD56^bright^NK cells as compared to controls, and decreased the percentage of mature CD56^+^ NK cells. We further demonstrated that lnc-CD56 knockdown reduced CD56 transcription. This evidence strongly suggested that lnc-CD56 functions as a positive regulator of CD56.

However, the possibility remains that lnc-CD56 is also important for the differentiation of precursors through the NK cell lineage. Because of the diversity of lncRNA functions, it is difficult to define the function(s) of a specific lncRNA. Like most lncRNAs, we suspect that lnc-CD56 also regulates its neighboring genes at the transcriptional or post-transcriptional level. lncRNAs can reportedly recruit RNA binding proteins to regulate protein-coding gene expression [41], and lnc-CD56 may function in this way. We used *catRAPID* to predict interactions between lnc-CD56 and NK-important transcription factors and found that TBX21, IRF2, IKZF2, ELF4 and EOMES may interaction with lnc-CD56. These transcription factors may be recruited by lnc-CD56 to promote CD56 expression and NK cell development. Thus, additional studies of lnc-CD56 in human NK cells will be required to unequivocally confirm the roles for this lncRNA in NK cell biology.

Collectively, the present lncRNA array-based study constitutes the first comprehensive inventory of lncRNAs in human NK cells. We identified lnc-CD56 as a novel positive regulator of CD56 in human NK cells. The novel, potentially functional lncRNAs identified in human NK cells in this study contribute to a more complete understanding of the molecular networks driving NK cell development and function.

## MATERIALS AND METHODS

### Samples

Healthy human peripheral blood samples were collected from the Hefei Blood Center. Umbilical cord blood and decidual samples were collected from the Hefei Maternal and Child Care Health Hospital after informed consent was obtained. All umbilical cord blood samples were from healthy, full-term newborns. All decidual samples were from healthy donors undergoing elective abortion in their first trimester between 6 and 12 weeks of gestation. All samples were approved by the Ethics Committee of the University of Science and Technology of China.

### Cell lines and human primary cells

Human embryonic kidney (HEK) 239T cells were cultured in DMEM high glucose medium (GE Healthcare Life Science) supplemented with 10% FBS and 100U/ml streptomycin/penicillin. Human primary cells were isolated as previously described [11, 12]. Cell purity was > 95% as determined by post-FACS analysis (Figure S1).

### Generation of human umbilical cord blood/CD34+ cell-derived NK cells

Umbilical cord blood samples were diluted 1:1 in PBS. Mononuclear cells were isolated using Ficoll-Hypaque (Solarbio) density gradient centrifugation according to the manufacturer's instructions. A Human CD34 MicroBead Kit (Miltenyi Biotec) was used to isolate CD34^+^ cells from umbilical cord blood mononuclear cells. Human umbilical cord blood/CD34^+^ cell-derived NK cells were prepared as previously described [12].

### LncRNA microarray analysis and computer analysis

For transcriptome profiling analysis, lncRNA and mRNA microarray hybridization in human decidual NK cells, peripheral blood NK cells, umbilical cord blood NK cells and peripheral blood T cells was performed using an Agilent Array platform (Agilent Technologies). During array hybridization, three donors were mixed into a single pool for each of dNK cells, cNK cells, pNK cells and T cells to balance individual differences. Heat map and red-blue color scales for differentially expressed lncRNAs and for lncRNA expression correlation analysis were exported using MEV 4.8.1 software. lncRNA-mRNA interactions were predicted using the database of Shanghai Biotechnology Corporation (SBC; http://www.shbio.com/sas.html), and mRNA-mRNA interactions were predicted using STRING [42] (http://string-db.org). lncRNA-mRNA interaction networks were exported using Cytoscape v3.1.0 software.

### RNA isolation and quantitative real-time PCR (qRT-PCR) analysis

Total RNA was extracted with TRIzol reagent (Invitrogen), and RNA reverse transcription was performed with Moloney Murine Leukemia Virus Reverse Transcriptase (Invitrogen) according to the manufacturer's protocol. Real-time PCR was conducted with SYBR Premix Ex Taq II (TaKaRa) on the Rotor Gene 3000 instrument according to the manufacturer's protocol. Primers are listed in Table S1. Primer sequences were synthesized by Sangon Biotech (Shanghai, China) .18S rRNA was used as the internal control.

### Plasmid construction

Short hairpin (sh)RNA sequences (Table S2) targeting lnc-CD56 and a negative control sequence (shNC) were synthesized by Sangon Biotech and then cloned into retrovirus shRNA expression vector pMSCV or lentivirus shRNA expression vector pLKO.1 (Addgene).

### Nucleofection and liposome transfection

Small interfering (si)RNA sequences (Table S3) targeting lnc-CD56, and a negative control sequence (siNC), were synthesized by GenePharma. An Amaxa Human NK cells Nucleofector Kit (VPA-1005) was used for human decidual NK cells. The pMSCV shRNA expression vector was transfected into 293T cells using the Lipofectamine 3000 Transfection Kit (Invitrogen).

### Lentivirus production and transduction

To produce lentiviral particles, pLKO.1-shRNA, pRRE, pREV and pVSV-G were co-transfected into 293T cells using the Lipofectamine 3000 Transfection Kit (Invitrogen). CD34^+^ cells were isolated from umbilical cord blood and incubated with multiple cytokines for 7-10 d as described above. After incubation, cells were spin-infected with lentiviral supernatant supplemented with polybrene (5ug/ml) at 400g for 2 h at 20°C.

### Flow cytometry

The following antibodies (BD Pharmingen) were used: Alexa 488-conjugated anti-CD56, PE-conjugated anti-CD34, Alexa 647-conjugated anti-CD56, anti-CD3 conjugated with Percp-cy5.5 and 7-AAD. Cell surface receptor staining was performed according to the manufacturer's instructions.

### Nuclear run-on assay

Experiments were performed as previously described [43, 44] in HEK-293T cells.

### Statistical analysis

Unless otherwise noted, data were expressed as the mean ± SEM of three or more independent experiments. Unless otherwise indicated, significance was determined by two-sided Student's *t*-test using GraphPad Prism Software (La Jolla, CA, U.S.A). *P* < 0.05 was considered significant.

## SUPPLEMENTARY MATERIALS FIGURES AND TABLES


