# Supplementary material for: A long noncoding RNA positively regulates CD56 in human natural killer cells
Source: Oncotarget. 2016 Oct 4;7(45):72546–58. doi: 10.18632/oncotarget.12466 (PMC5341928; doi:10.18632/oncotarget.12466)
Supplement: Supplementary file 1 [file oncotarget-07-72546-s001.pdf]

A long noncoding RNA positively regulates CD56 in human natural killer cells

Supplementary Material

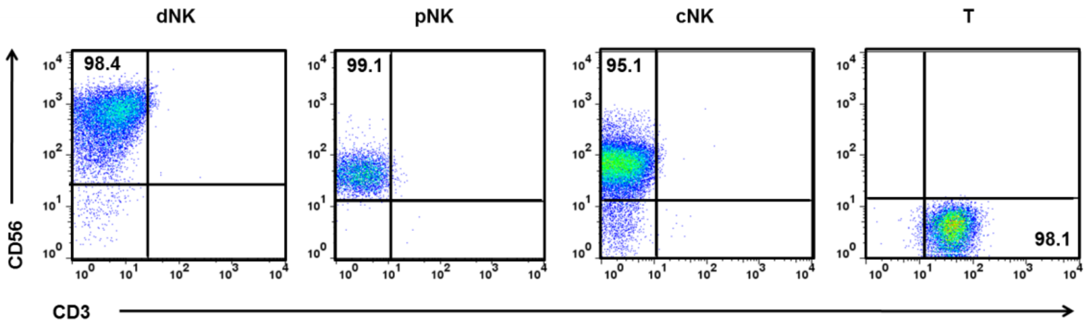

Supplementary Figure 1: Human primary NK cell and T cell purities. Human decidual NK cells were isolated from healthy donor decidual samples. Human cord blood NK cells were isolated from healthy donor umbilical cord blood samples. Human peripheral blood NK and T cells were isolated from healthy donor peripheral blood samples. NK and T cell isolations were conducted as previously described [11]. Isolated NK and T cell purities were analyzed using anti-CD3 conjugated with Percp-cy5.5 and anti-CD56 conjugated with PE via flow cytometry.

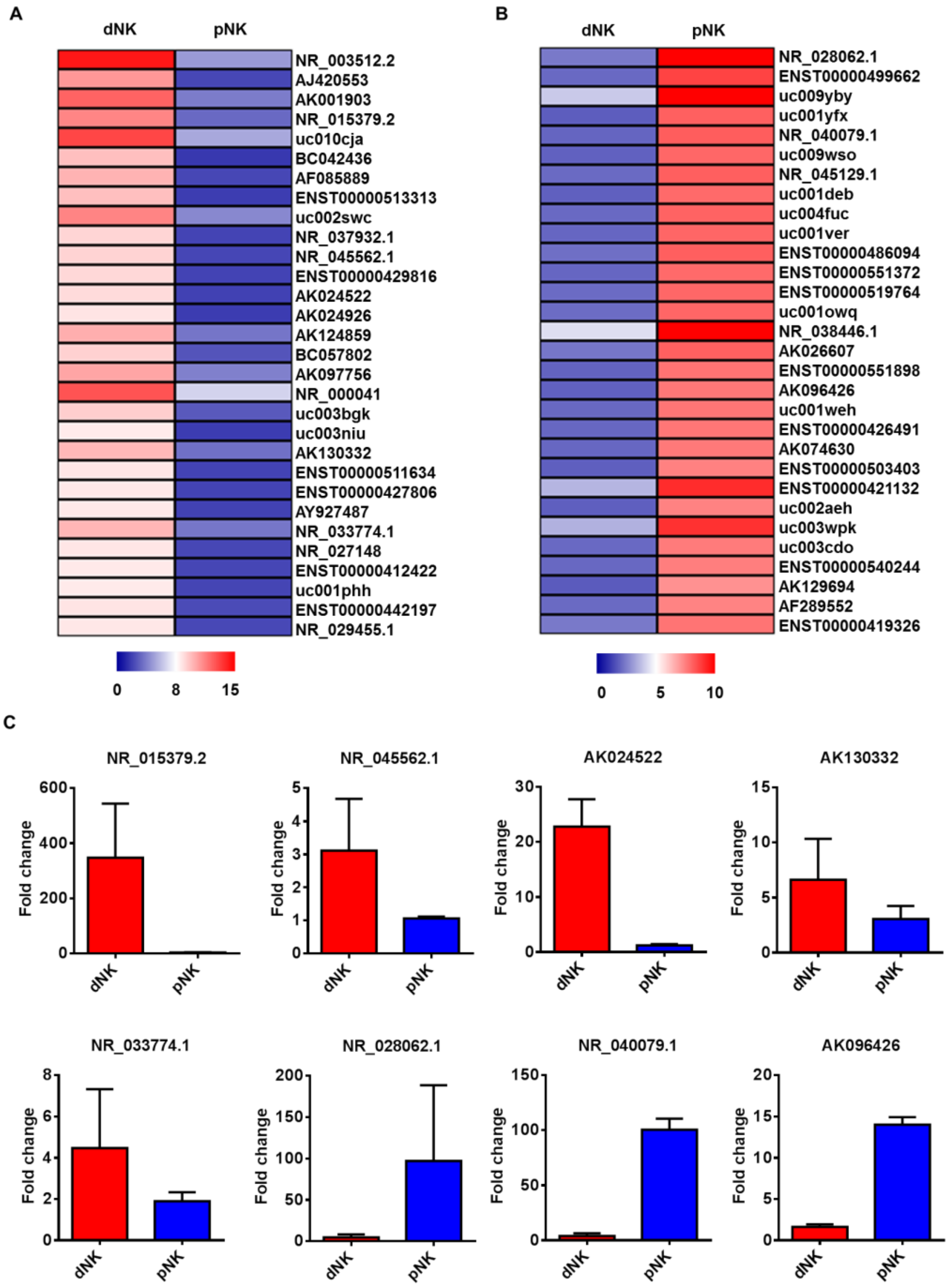

Supplementary Figure 2: LncRNA profiles in dNK and pNK cells. Heat map of the top 30 lncRNAs ranked by fold change that were up- or downregulated in human dNK (relatively immature NK) compared to pNK (relatively mature NK) cells with more than a two-fold change are shown in **A.** and **B.** respectively. **C.** qRT-PCR verification of randomly selected lncRNAs in human dNK and pNK cells. Data represent three independent experiments.

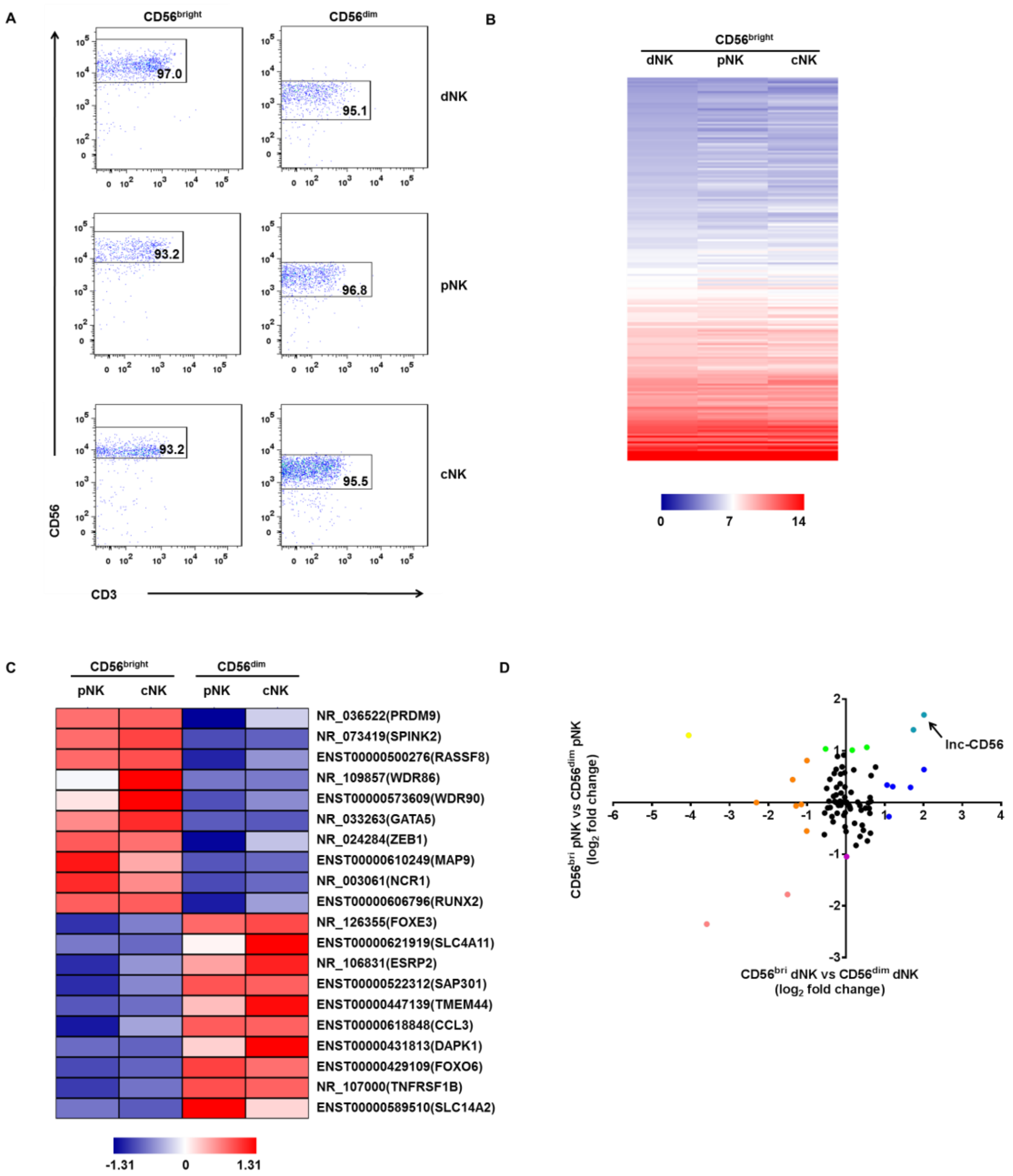

Supplementary Figure 3: lncRNA profiles in CD56^{bright} and CD56^{dim} dNK, pNK and cNK cells. Purity of human primary CD56^{bright} dNK, CD56^{dim} dNK, CD56^{bright} pNK, CD56 ^{dim} pNK, CD56^{bright} cNK and CD56^{dim} cNK cells **A.** Human decidual, cord blood and peripheral blood monocytes were isolated as previously described [11]. CD56^{bright} and CD56^{dim} NK cells were purified using anti-CD3 conjugated with APC-Cy7, anti-CD45 conjugated with PE and anti-CD56 conjugated with 647 via flow cytometry. Heat maps of lncRNAs in human CD56^{bright} dNK, CD56^{bright} pNK

and CD56^{bright} cNK cells **B.** and lncRNAs with predicted target genes involved in NK cell differentiation, cytokine secretion and cytotoxic activity **C.** Displayed lncRNAs were up- or downregulated at least 2-fold in CD56^{bright} pNK cells and CD56^{bright} cNK relative to CD56^{dim} pNK cells and CD56^{dim} cNK cells. Scatter plots of log base 2 fold changes of lncRNAs targeting clusters of differentiation (CD) molecules in different cell types **D.** Each plot represents one lncRNA. Each color represents one expression type.

SUPPLEMENTARY TABLES

Supplementary Table 1: lncRNA primer sequences.

gene	P1	P2
AK001903	TGCGCAGTAGCAAAGATGGT	CAACCCCCATGTTATCCCCG
AK129667	ACGTCCCAAGCCCTAAATGG	GCGGCTGAAGAGAGAACCTT
NR_024074	TGCTTCCTGCCTCTGACTTT	CCACTGTCTCTTCCCCTGTGA
AK129737	GAGGTGGGGTGTCTTTTCGT	TCAGCCTCCTCTCAGGTTGT
NR_033766.1	TGTGCAGAGCAGAGACGTAA	CACCTGGGGAGTCATCATGG
BC042436	GAGCCAAAGTGGCATCTCCT	AGGCAGAGAGACACGCTCTA
AF085889	AGTCTGTCACCCACAAAGGG	GGTGTGTGAACCTTCTCGCT
BC057802	AACGGGACGTTTCGTTCTTC	GGAAGAAAGTTGGGAGCGGT
NR_038446.1	GCTGTTGCCCCACTCCTATT	TCCAATAAGGCAGCACTGGG
BC045184	TGGGTGATGGGGAGATAGCA	TGCAGCATCCATCAAGGGTT
uc003ebh(TIGIT)	TTTGTTGTGGGTGACGAGTT	GGCGAAACCATCTTGATCCG
AX794816(ITGAL)	ATGGTAGTGGCTGAGTTGTC	CTGTCCAGGGCATTATCTTG
AK090692(FYN)	CACAGACCAGCATAACAGTG	GAAGAAGAAACAGGCTCAGA
BC020376(UNC13D)	CCGCAGCCTCACAAAGACCT	GCTCACCGTGCTGGACTACG
AK074630(PTK2B)	CTCCTTTGGTGCTGAACTGC	AAGGCACGACATCTGAAAAC
AK096651(CD160)	GGGACGACTTATAGGATAGA	CAGCCACTTGTGATACTTAC
AK124429(IL7R)	AAAGCAGAAGTTCCATGTCTGAAC	GCCAATGCTGCTTCTGTGTG
AK054790(GRB2)	ACCGATTCTCCTTCCTCAGC	AGTAACTCATGCCCGTAATC
uc001muq(CD59)	AGAGTCCAGATGACCCAAAGGG	CCCGACCCGAAATGTAGCCTGG
AK123812(SOCS2)	CGACAGAGGGAGACTTGGTCAT	TTCTGGGTGAGGGACTGGGATA
AK024994(CCL3/CCL4)	AGATGGCTCTATACGATAAG	TGGTCTCCTAGTAGGGAATG
AF088007(PRKCQ)	CCTTGGATTGGCTCATCTGC	AACATCTGCCTTCTGGGTGA
uc001dkx(BCL10)	CCTCCTGAGACCTACACCAC	ATCGGATTCTGACTTTCCTT
uc002rjt(UCN)	GGGTGGTTTGGGTCTATTTGG	CCCTGACGGTTCATTTCAGA
AK123278(ADAM17)	TGCAATGGAATTTACCCAGC	GAGCAACCACTAACCACCCT
AK001094(ENG)	GGCAGGGGAACAAAGAGCGT	GCAAGGAGGCAGCAAGAAGT
AK129712(SMAD3)	CATCTGTGGCTCAAATCAAG	AACCCAAGTTCCTCTAAATC
NR_003512.3(IGF2/IL6)	GCAGCCTTTGTGAACCAACA	AAGGCTCTCTGCCGAAACTG
NR_045484.1(BCL10)	GCCAATGTTTAAGAAACGGG	TCAGTAAATCGGATGGAAGA
NR_033994.1(TNFSF10)	TCAGCTCGTTAGAAAGAAAAGCA	TGCTCAGGAATGAATGCCCA
CR936696(IL1R1)	AGTATGCCTTCTGGCAGTAACA	ATTGCAAGGGAACCGTCACT
AK130588(BCL11B)	TGATTCTCAAGGAAGGGATC	AGAGCAGACTCACGCACCCA
AK129694(PRKCB)	GAGGAGAAGGGACTCTATAT	TCAGGACCACTTATTCAAAG
ENST00000518993(TOX)	TGCTTGGTGCTCCGAAATGC	GGGCAGTGTCCTTGGTGAGA
NR_036634.1(TGFBR3)	AGGTGATGTTTCCGTGGGGC	GGGGACAGTAGTGGTTGGCC
AK098125(TCF7L1)	GCTGCCTGGGTGAGATGCTG	TCTGCCTTTGCTACTTCCTG
uc002flc(IL17C)	TGTTGCCAAGTGCTCATCTG	CGTGGGAGAAAGCCTAGTGC
AF085950(TGFBR2)	CCAAATGCCTTCACTCTGCC	GCATGTGACTTACGCTCACC
AK090690(CD247)	AGAAATGCTGGTGCTGATGG	CTCTATGGTCTGGCTCGTCT
AB073357(GATA3)	GTTCCAGCTTGCTATTTTTG	CTATGAGTCTTCCCTTCGGT
AJ005814(HOX)	AAGTAGACAGGGCACTCGTTA	GCTGGAAAGGTCGGTTGTAA
BC026731(EPAS1)	ACAACTGCGATGCCTATCGG	AGTCCGCTAACGAACCAGGT
lnc-CD56	GAGAAGTATGGCGGACGGTT	CTTTGGCGAGTGCCTTTCTG
CD56	CTGCGAGGTATTTGCCTATCC	CCTGGGCTGTGCTGGAGTATG
TBX21	CTGGAGGTGTCGGGGAAAC	ATGGGAACATCCGCCGTCC
IRF2	GACAGTCCCATCTGGACAGC	TCAGTCGTTTCGCTTTCTGC
IKZF2	GCTCCTCGCTGAAGATGGAG	GGTGACAATGTCGGGCTCA
ELF4	TCCACTTCTCCTTTCGCCG	AACCACAGGAGCGACCTGA
EOMES	GGGTTCCAGGTTCTGGCTTC	AAAGGAAACATGCGCCTGCC
18S rRNA	CAGCCACCCGAGATTGAGCA	TAGTAGCGACGGGCGGTGTG

Supplementary Table 2: shRNAs targeting lnc-CD56.

shNC	CCGTTCTCCGAACGTGTCACGT
shlnc-CD56-1	ATCCCATTCACTCCTTGGAATG
shlnc-CD56-2	AGGAGACACAGTCTTGCTGAAT

Supplementary Table 3: siRNAs targeting lnc-CD56.

	sense（5'-3'）	antisense（5'-3'）
siNC	UUCUCCGAACGUGUCACGU dTdT	ACGUGACACGUUCGGAGAA dTdT
silnc-CD56-1	CCAUACAUAGAGUCACAUU dTdT	AAUGUGACUCUAUGUAUGG dTdT
silnc-CD56-2	GGGAGAGAUGAUUCCUGAU dTdT	AUCAGGAAUCAUCUCUCCC dTdT
